# Sedentary Behavior and Associated Factors Among Working Adults in Eastern Ethiopia

**DOI:** 10.3389/fpubh.2021.693176

**Published:** 2021-09-07

**Authors:** Aboma Motuma, Tesfaye Gobena, Kedir Teji Roba, Yemane Berhane, Alemayehu Worku

**Affiliations:** ^1^School of Nursing and Midwifery, College of Health and Medical Sciences, Haramaya University, Harar, Ethiopia; ^2^Department of Environmental Health Science, College of Health and Medical Sciences, Haramaya University, Harar, Ethiopia; ^3^Department of Epidemiology and Biostatics, Addis Continental Institute of Public Health, Addis Ababa, Ethiopia; ^4^Department of Epidemiology and Biostatistics, School of Public Health, Addis Ababa University, Addis Ababa, Ethiopia

**Keywords:** high, prevalence, sedentary behavior, working adults, Ethiopia

## Abstract

**Background:** Sedentary behavior is a major risk factor for non-communicable diseases. Due to changes in lifestyle, sedentary behavior is increasing in sub-Saharan Africa. However, information on the extent of sedentarism among various segments of the population is scant in low-income countries. The objective of this study was to assess the extent of high sedentary behavior and associated factors among working adults in eastern Ethiopia.

**Methods:** A crosssectional study was conducted among 1,164 working adults at Haramaya University from December 2018 to February 2019. Data were collected through face-to-face interviews using the WHO STEPS and sedentary behavior questionnaire. All reported sedentary activities were added to calculate the total number of hours spent on sedentary behavior, which was then dichotomized into two categories. Those who had ≥8 sedentary hours per day were categorized as having high sedentary behavior. The prevalence ratio (PR) with 95% confidence intervals (CIs) was calculated. Factors associated with outcome variables were identified using Poisson regression with a robust variance statistical model.

**Results:** The prevalence of high sedentary behavior was 20.3% (95% CI, 18.0–22.7%) among the study participants. The prevalence of high sedentary behavior was associated with age 45–54 years adjusted PR (APR: 2.00; 95% CI = 1.01–3.97) and 55–64 years (APR: 2.16; 95% CI = 1.03–4.57), being a non-manual worker (APR: 2.11; 95% CI = 1.46–3.05), frequent khat chewers (APR: 1.57; 95% CI = 1.22–2.01), with body mass index of ≥25 kg/m^2^ (APR: 1.93; 95% CI = 1.53–2.44), and regular alcohol drinker (APR: 1.39; 95% CI = 1.11–1.76).

**Conclusion:** One-fifth of working adults had high sedentary behavior. Factors associated with high sedentary behaviors were older age, being a non-manual worker, substance-use behaviors, and having a high body mass index.

## Introduction

Sedentary behavior is characterized by low energy expenditure (1), whether that behavior is due to leisure or occupation (2). The increasing availability of modern technology, transportation, and communication systems have exacerbated the problem in low-income countries (3). Sedentary behavior has emerged as a public health concern due to the increasing trend in non-communicable diseases (NCDs) (4) with grave consequences of morbidity, death, and disability (5–8).

Sedentary behavior has an important implication on the health status of working adults (9). Evidence shows that white collar workers such as university employees accumulate a high sedentary time at workplaces (10), which often extends to outside normal working hours (11, 12). Studies have shown that university workers spend over two-thirds of their workday sitting (9, 13, 14). Studies conducted among college employees also indicated that the working days of the administrative staff were more sedentary when compared with faculty members (15). Evidence shows that high sedentary behaviors were associated with metabolic syndrome (16) and obesity (17) among university faculty.

Evidence indicates that occupational sedentary behavior was not significantly associated with productivity (9); however, employees with increased sedentary behavior were more prone to illnesses such as back pain, mental health, diabetes, and cardiovascular diseases, which indirectly can influence productivity at work (12, 18). Generally, high sedentary behavior is one of the modifiable risk factors contributing to premature mortality among adults in low-income countries (6, 19), although sedentarism is still is relatively low in the general population compared with higher-income countries (20).

However, this difference is rapidly closing in some occupational categories such as that of university employees (21). The risk factors for high sedentary behavior have been well-studied in high and middle-income countries. The information on the extent of and risk factors associated with high sedentary behavior is scant in the sub Saharan Africa. Therefore, the aim of this study was to assess the prevalence of high sedentary behavior and its associated factors among working adults in Eastern Ethiopia.

## Materials and Methods

### Study Setting, Design, and Period

This study was conducted at a public university (Haramaya University) found in East Ethiopia. Haramaya University has four campuses, eleven academic and research units, and four health clinics that provide services to the university community. The university also runs a specialized referral hospital in Harar town that provides services to the general population. The total student population in both regular program and non-regular programs is 30,355. The students attend their education at the undergraduate and graduate levels. The university has about 7,176 employees, with a gender mix of 28.1% female and 71.9% male, and a job mix of 21.1% academic and 78.9% administrative staff. This university was considered for the study because of its large employee size and diversity of jobs, which ranges from senior academic positions to manual work. The university's main campus is located in a rural setting where khat (a locally cultivated stimulant plant) production and consumption is high. A crosssectional study was conducted among 1,164 working adults from December 2018 to February 2019.

### Study Population and Sampling Procedures

The study involved all permanent employees who worked for at least 6 months at the university. All departments and units of the university were considered for the study. The study participants were selected from each unit proportional to the size of their respective department staff size. In each unit, the study participants were selected randomly from the payroll roster, which was used as the sampling frame. We calculated the sample size assuming a 29.6% prevalence of sedentary behavior (22), 95% level of confidence, 3% margin of error, and 20% non-response rate. Accordingly, the calculated sample size was 1,106. The total sample size was distributed to each unit as described above.

### Study Tool and Data Collection Procedures

Data were collected using a structured questionnaire adapted from the WHO STEPS and sedentary behavior questionnaires (23, 24). The questionnaire was previously used in Ethiopia in various settings. The English questionnaire was translated to Amharic by language experts who are competent in both English and Amharic to ensure the consistency and accuracy of the translation. Then, the tool was pretested in a nearby public university. Some questions and the translation were refined based on the feedback we obtained during the pretest.

### Variables and Measurement

High sedentary behavior was measured for 10 activities: watching television, playing computer/video games or social media, sitting during eating and drinking, sitting while listening to music, sitting and talking on the phone, doing paperwork or office work, napping, sitting and reading, sitting for socializing with friends and/or family activities, and sitting and driving/riding in a car or bus (24). The items were completed for weekdays and weekends separately and grouped into three domain-specific (occupation, leisure time, and transportation) sedentary behavior. The occupation domain included paperwork or desk-based office work; leisure time domain comprised watching television, napping, playing computer/video games, sitting during eating and drinking, sitting while listening to music, sitting and talking on the phone, sitting during reading, and sitting for socializing with family or/and friends; and transportation domain comprised of sitting and driving motorized vehicle (1, 6).

The average total sedentary time per day was obtained by computing the sum of the 10 items. Currently, there is no well-accepted threshold to categorize a cut-off point for high sedentary behavior (25). However, previous evidence indicated that 8 or more hours of sedentary behavior was associated with a high risk for premature mortality (26–30). Hence, we used ≥8 h/day as a cut-off point for high sedentary behavior (6, 26, 27). For the specific domains (occupation, transportation, and leisure) a 75th percentile or above was used as a thresholds cut-off of high occupation sedentary behavior ≥3 h/day, high leisure sedentary behavior ≥5.05 h/day, and high transportation sedentary behavior ≥1 h/day (6).

The age of respondents was recorded based on their response and categorized into 18–24 years, 25–34 years, 35–44 years, 45–54 years, and 55–64 years of age groups. Education level was grouped into ≤ grade 8, grade 9–12, college and undergraduate, and postgraduate. The working status of the respondent was grouped into manual/laborer work or non-manual based on the classification of the Human Resource Office of the university. The income cut-off of the study participants was set to roughly correspond to the poverty line in accordance with the World Bank (31).

Body weight was measured using a digital weighing scale to the nearest 0.1 kg with the participants barefooted and wearing light clothes. Height was measured using a stadiometer to the nearest 0.1 cm with the shoes and any hats or hair ornaments of the participant removed. Body mass index was calculated as weight in kilograms over height in meters squared, and was categorized according to World Health Organization (WHO) criteria (underweight: BMI < 18.5 kg/m^2^; normal: BMI = 18.5–24.9 kg/m^2^; overweight: BMI = 25.0–29.9 kg/m^2^; and obese: BMI ≥ 30.0 kg/m^2^) (23).

Smoking status was categorized as never smoked, former smoker, and current smoker. Alcohol consumption was measured based on the WHO Stepwise approach. In this study, we asked about the drinking habits of the participant. The alcohol drinking habits of the study participants during the survey period were classified as never, occasional, and frequent drinkers (23). Khat (catha edulis) use habit was grouped into never, the occasional, and habitual (frequent) user. The total physical activity score was computed as the sum of all metabolic equivalent (MET)-minutes per week for vigorous intensity physical activity, moderate intensity physical activity, and walking. The sum MET-minutes per week was categorized as high (3,000 MET-minutes or above), moderate (between 2,999 and 600 MET-minutes), and low (<600 MET-minutes) (32). Data collection methods and study procedures have been presented in detail in a previous publication (33).

### Data Management and Analysis

The completed questionnaires were double entered into EpiData Version 3.1, cleaned, and exported to STATA 14 statistical software for data analysis. The missing values of each variable were <1%, and the chance of missing was unrelated to any of the variables and considered as missing completely at random. Hence, a complete case analysis was used to handle the missing data. Descriptive statistics were used to calculate the frequency distribution, proportions with 95% confidence interval for categorical variables, and mean and standard deviation for continuous variables. A Poisson regression model with robust variance estimation was used to assess factors associated with the outcome variable. The Poisson regression analysis model is an appropriate analytical model for estimating the prevalence ratio (PR) in crosssectional studies binary and common outcomes (34). The backward regression was fitted with selected explanatory variables. Crude PRs (CPRs) with 95% CIs were estimated to assess the association between each independent variable and the outcome variable. Variables with a *p*-value of ≤0.25 in the bivariate analysis were considered for multivariable analysis. A *p*-value of <0.05 was used to declare the level of statistical significance in the multivariable analysis, and adjusted PRs (APRs) along with 95% CIs were estimated. The explanatory variables were tested for multicollinearity and checked for interaction among the explanatory variables before fitting them into multivariable model.

## Results

### Socio-Demographic Characteristics

A total of 1,200 study participants were invited for the study and 1,164 study subjects actually participated in the study, which yields a response rate of 97%. Nearly half (51.4%) of the participants were males and the mean age of the study participants was 35.5 years. The proportion of currently married was 57.3%, diploma or higher degree holders was 63.5%, and those with 10 years or below at the university were 76.1% (Table 1).

**Table 1:** Socio-demographic characteristics of the study participants in Eastern Ethiopia, 2019 (*n* = 1,164).

**Variables**	**Category**	**Number**	**Percent**
Sex			
	Male Female	598 566	51.4 48.6
Age group in years			
	18–24 25–34 35–44 45-54 55–64	80 537 324 151 72	6.9 46.1 27.8 13.0 6.2
Occupation			
	Manual/laborer work Non-manual	409 755	35.1 64.9
Level of education			
	Primary school Secondary school College/undergraduate ≥ Second degree	193 232 559 180	16.6 19.9 48.0 15.5
Service years			
	<5 years 5–10 years 10.1–15 years >15 years	492 394 148 130	42.3 33.8 12.7 11.2
Marital status			
	Single Married Divorced/widowed	427 667 70	36.7 57.3 6.0

### Lifestyle and Health Related Characteristics

About half of the study participants around 571 (49.1%) had low physical activity (<600 MET-minutes per week), 396 (34.0%) were regular khat users, and 553 (47.5%) were frequent alcohol consumers. Few of the study participants 131(11.3%) were ever smokers of whom 9.4% were males. Out of these, 59 (5.1%) were current smokers. Four hundred and thirteen (35.5%) and 109 (9.8%) working adults were overweight and obese, respectively. Most of the study participants 512 (44%) reported their health status rank from fair to poor health status (Table 2).

**Table 2:** Lifestyle and health related characteristics of study participants in Eastern Ethiopia, 2019 (*n* = 1,164).

**Variables**	**Category**	**Number**	**Percent**
Smoking status			
	Never smoked	1,033	88.7
	Former smoker	72	6.2
	Current smoker	59	5.1
Drinking habit			
	Not at all/occasional	611	52.5
	Frequent	553	47.5
Khat-chewing habit			
	Never/occasional	768	66.0
	Frequently	398	34.0
BMI in kg/m^2^			
	<18.5	113	9.7
	18.5–24.9	638	54.8
	25–29.9	305	26.2
	≥30.0	108	9.3
Physical activity
	<600 MET	571	49.1
	600–2,999 MET	367	31.5
	≥3,000 MET	226	19.4
Self-reported health status			
	Fair/poor	512	44.0
	Excellent	652	56.0

*BMI, body mass index; kg/m^2^, kilogram per meter square; MET, metabolic equivalent*.

### The Mean Self-Report Sedentary Behavior Among Study Participants

The total mean (±SD) time of sedentary behavior was 5.9 (±2.1) hours per day, 6.2 (±2.2) on weekends, and 5.8 (±2.3) hours per day on working days. The mean (±SD) sedentary time reported during leisure time was 3.7 (±1.5) hours per day, 1.9 (±1.8) hours per day at workplace, and 0.4 (±0.5) hours per day while using transportation (Table 3). The commonest sedentary activities included watching TV/video or playing social media, doing paperwork or desk-based office work, and socializing with friends and/or family. Almost all sedentary time spent on different activities showed a slight increase on weekends compared with weekdays (Table 4).

**Table 3:** The mean (±SD) sedentary behavior by specific domain in hours per day among the study participant's characteristics in Eastern Ethiopia, 2019.

**Variables**	**Occupation sedentary time**	**Leisure sedentary time**	**Transport sedentary time**	**Total sedentary time**
Mean (±SD)	1.9 (±1.8)	3.7 (±1.5)	0.4 (0.5)	5.9 (±2.1)
Age				
18–24	1.80 ± 1.61	3.44 ± 1.44	0.25 ± 0.20	5.52 ± 1.93
25–34	2.10 ± 1.78	3.56 ± 1.35	0.37 ± 0.39	5.93 ± 1.94
35–44	1.76 ± 1.86	3.76 ± 1.51	0.41 ± 0.55	5.89 ± 2.17
45–54	1.53 ± 1.87	3.94 ± 1.51	0.35 ± 0.47	5.82 ± 2.34
55–64	1.38 ± 1.64	4.07 ± 1.72	0.56 ± 0.88	6.05 ± 2.30
Gender				
Male	2.27 ± 1.90	3.67 ± 1.46	0.45 ± 0.59	6.23 ± 2.05
Female	1.43 ± 1.59	3.71 ± 1.46	0.31 ± 0.32	5.51 ± 2.05
Occupation category				
Manual work	0.81 ± 1.20	3.51 ± 1.50	0.28 ± 0.27	4.86 ±1.93
Non-manual	2.43 ± 1.83	3.79 ± 1.43	0.43 ± 0.56	6.43 ± 1.95
Khat chewing habit				
No/occasional	1.92 ± 1.83	3.61± 1.39	0.36 ± 0.43	5.84 ± 2.00
Regular	1.63 ± 1,74	3.85 ± 1.58	0.43 ± 0.58	5.97 ± 2.24
Reported physical activity				
Low	1.98 ± 1.79	3.77 ± 1.39	0.39 ± 0.51	6.06 ± 1.97
Moderate	1.88 ± 1.81	3.61 ± 1.57	0.37 ± 0.46	5.82 ± 2.13
High	1.53 ± 1.83	3.64 ± 1.44	0.35 ± 0.48	5.53 ± 2.24

*(±SD), standard deviation*.

**Table 4:** Self-report sedentary time by doing different activities among study participants in Eastern Ethiopia, 2019.

**Sedentary activities**	**Mean (±SD) weekdays (h/day)**	**Mean (±SD) weekends (h/day)**
Watching TV/video	1.17 (±0.96)	1.05 (±1.07)
Listening to music	0.46 (±0.57)	0.61 (±0.66)
Playing computer or mobile game	0.11 (±0.29)	0.10 (±0.25)
Talking on the phone	0.28 (±0.29)	0.33 (±0.34)
Eating or drinking	0.55 (±0.37)	0.74 (±0.38)
Doing paperwork or office work	1.86 (±1.81)	0.47 (±0.74)
Napping	0.17 (±0.27)	0.27 (±0.37)
Reading	0.39 (±0.62)	0.42 (0.67)
Socializing with friends and/ or family	0.63 (±0.59)	1.01 (±0.71)
Driving or riding in car or bus	0.39 (±0.57)	0.33 (±0.41)

*(±SD), standard deviation; TV, television; h/day, hour per day*.

### Prevalence of High Sedentary Behavior

The prevalence of high sedentary behavior was 20.3% (95% CI: 18.0, 22.7) among study participants. The prevalence of high sedentary behavior was higher among males (23.8%) compared with females, (16.6%) (Table 5). Based on the cut-off value 75th percentile, the prevalence of high occupation sedentary behavior (≥3 h per day) was 25.6%, the prevalence of high leisure sedentary behavior (≥5.05 h per day) was 18.8%, and the prevalence of high transportation sedentary behavior (≥1 h per day) was 7.9% (Table 6).

**Table 5:** Prevalence of high sedentary behavior by characteristics of study participants in Eastern Ethiopia, 2019 (*n* = 1,164).

**Variables**	**Category**	**SB (≥8 h/day)**		**No SB (<8h/day)**	
		**Number**	**Percent**	**Number**	**Percent**
Total sedentary time		236	20.3	928	79.7
Sex					
	Male	142	23.8	456	76.2
	Female	94	16.6	472	83.4
Age in years					
	18–24	9	11.3	71	88.7
	25–34	93	17.3	444	82.7
	35–44	74	22.8	250	77.2
	45–54	41	27.2	110	72.85
	55–64	19	26.4	53	73.6
Educational level					
	≤ Grade 8	28	14.5	165	85.5
	Grade 9–12	38	16.4	194	83.6
	Diploma/degree	104	18.6	455	81.4
	Masters and above	66	36.7	114	63.3
Marital status					
	Never married	78	18.3	347	81.7
	Married	143	21.4	524	78.6
	Divorced/widowed	15	21.4	55	78.6
Occupation category					
	Manual work	47	11.5	362	88.5
	Non-manual	189	25.0	566	75.0
Khat chewing habit					
	Never/occasional	130	16.9	638	83.1
	Frequently	106	26.8	290	73.2
Drinking habit					
	Not at all/occasionally	93	15.2	518	84.8
	Frequently	143	25.9	410	74.1
BMI in Kg/m^2^					
	<18.5	3	2.7	110	97.3
	18.5–24.9	104	6.3	534	83.7
	25–29.9	85	27.9	220	72.1
	≥30	44	40.7	64	59.3

*BMI, body mass index; kg/m^2^, kilogram per meter square; SB, sedentary behavior; h/day, hour per day*.

**Table 6:** Selected characteristics of participants by specific domain occupation, leisure, and transport sedentary time among working adults in Eastern Ethiopia, 2019.

**Variables**	**Occupation sedentary time**		**Leisure sedentary time**		**Transport sedentary time**	
	**≥3 h/day**	**<3 h/day**	**≥5.05 h/day**	**<5.05 h/day**	**≥1 h/day**	**<1 h/day**
	**No (%)**	**No (%)**	**No (%)**	**No (%)**	**No (%)**	**No (%)**
Domain-specific	298 (25.6)	866 (74.4)	219 (18.8)	945 (81.2)	92 (7.9)	1,072 (92.1)
Gender						
Male	205 (34.3)	393 (65.7)	109 (18.2)	489 (81.8)	62 (10.4)	536 (89.6)
Female	93 (16.4)	4739 (83.6)	110 (19.4)	456 (80.6)	30 (5.3)	536 (94.7)
Age in years						
18–24	16 (20.0)	64 (80.0)	10 (12.5)	70 (87.5)	1 (1.3)	79 (98.7)
25–34	159 (29.6)	378 (70.4)	84 (15.6)	453 (84.4)	38 (7.1)	499 (92.9)
35–44	78 (24.1)	246 (75.9)	73 (22.5)	251 (77.5)	34 (10.5)	290 (89.5)
45–54	30 (19.9)	121 (80.1)	32 (21.2)	119 (78.8)	10 (6.6)	141 (93.4)
55–64	15 (20.8)	57 (79.2)	20 (27.8)	52 (72.2)	9 (10.5)	63 (87.5)
Educational level						
≤ Grade 8	6 (3.1)	187 (96.9)	36 (18.6)	157 (81.4)	18 (9.3)	175 (90.7)
Grade 9–12	14 (6.0)	218 (94.0)	48 (20.7)	184 (79.3)	26 (11.2)	206 (88.8)
Diploma/degree	175 (31.3)	384 (68.7)	98 (17.5)	461 (82.5)	37 (6.6)	522 (93.4)
Masters and above	103 (57.2)	77 (42.8)	37 (20.6)	143 (79.4)	11 (6.1)	169 (93.9)
Marital status						
Never married	129 (30.2)	29 (69.8)	62 (14.5)	365 (85.5)	31 (7.3)	396 (92.7)
Married	162 (24.3)	505 (75.7)	143 (21.4)	524 (78.6)	57 (8.6)	610 (91.4)
Divorced	7 (10.0)	63 (90.0)	14 (20.0)	56 (80.0)	4 (5.7)	66 (94.3)
Occupation						
Manual work	23 (5.6)	386 (94.4)	68 (16.6)	341 (83.4)	18 (4.4)	391 (95.6)
Non-manual	275 (36.4)	480 (63.6)	151 (20.0)	604 (80.0)	74 (9.8)	681 (90.2)
Khat chewing						
No/occasional	199 (25.9)	569 (74.1)	124 (15.8)	647 (84.2)	50 (6.5)	718 (93.5)
Regularly	99 (25.0)	297 (75.0)	95 (24.8)	298 (75.2)	42 (10.6)	354 (89.4)
Drinking habit						
Never/occasional	116 (19.0)	495 (81.0)	103 (16.9)	508 (83.1)	51 (8.4)	560 (91.6)
Frequently	182 (32.9)	371 (67.1)	116 (21.0)	437 (79.0)	41 (7.4)	512 (92.6)
BMI in kg/m^2^						
<18.5	21 (18.6)	92 (81.4)	5 (4.4)	108 (95.6)	3 (2.7)	110 (97.3)
18.5–24.9	163 (25.6)	475 (74.5)	94 (14.7)	544 (85.3)	43 (6.7)	595 (93.3)
25.0–29.9	80 (26.2)	225 (73.8)	74 (24.3)	231 (75.7)	37 (12.1)	268 (87.9)
≥30.0	34 (31.5)	74 (68.5)	46 (42.6)	62 (57.4)	9 (8.3)	99 (91.7)

*BMI, body mass index; kg/m^2^, kilogram per meter square; h/day, hours per day; No, number; %, percent. The cut-point for occupation, leisure, and transport sedentary time is based on the 75th percentile*.

### Factors Associated With High Sedentary Behaviors Among Study Participants

The prevalence of high sedentary behavior was higher among the age group of 45–54 years (APR: 2.00; 95% CI = 1.01–3.97) and 55 and above years (APR: 2.16; 95% CI = 1.03–4.57) as compared with the age group 18–24 years. The prevalence of high sedentary behavior was higher among being a non-manual worker compared with manual workers (APR: 2.11; 95% CI = 1.46–3.05). The prevalence of high sedentary behavior was higher among individuals who frequently use/chew khat compared with those who never or occasionally chew khat (APR: 1.57; 95% CI = 1.22–2.01); among individuals who regularly drink alcohol compared with never or occasional drinkers (APR:1.39; 95% CI = 1.11–1.76); and among individuals who had a BMI 25 kg/m^2^ or above compared with those who had a BMI <25 kg/m^2^ (APR: 1.93; 95% CI = 1.53–2.44) (Table 7).

**Table 7:** Factors associated with high sedentary behavior among study participants in Eastern Ethiopia, 2019.

**Variables**	**High Sedentary time ≥ 8 h/day**		**CPR (95% CI)**	**APR (95% CI)**
	**Yes number (%)**	**No number (%)**		
Sex				
Male (ref)	142 (23.7)	456 (76.3)	1	1
Female	94 (16.6)	472 (83.4)	**0.69 (0.55–0.88)**	0.93 (0.70–1.122)
Age group in years 19-24 (ref)	9 (11.3)	71 (88.7)	1	1
25–34	93 (17.3)	444 (82.9)	1.53 (0.80–2.92)	1.29 (0.69–2.42)
35–44	74 (22.8)	250 (77.2)	**2.03 (1.06–3.87)**	1.64 (0.85–3.19)
45–54	41 (27.2)	110 (72.8)	**2.41 (1.23–4.71)**	2.00 (1.01–3.97)^*^
55–64	19 (26.4)	53 (73.6)	**2.34 (1.13–4.85)**	2.16 (1.03–4.57)*
Educational status				
Primary school (ref)	28 (14.5)	165 (85.5)	1	1
Secondary school	38 (16.4)	194 (83.6)	1.12 (0.72–1.76)	0.98 (0.62–1.55)
College/undergraduate	104 (18.6)	455 (81.4)	1.28 (0.87–1.88)	0.96 (0.61–1.51)
≥Second degree	66 (36.7)	114 (63.3)	**1.52 (1.70–3.74)**	1.50 (0.98–2.47)
Occupation category				
Manual work (ref)	47 (11.5)	362 (88.5)	1	1
Non-manual	189 (25.0)	566 (75.0)	**2.17 (1.62–2.92)**	**2.11 (1.46**– 3.05)^***^
Khat chewing habit				
No/ occasional (ref)	130 (16.9)	638 (83.1)	1	1
Frequently	106 (26.8)	290 (73.2)	**1.58 (1.26–1.98)**	**1.57 (1.22**–2.01)^***^
Body max index				
<25 kg/m^2^ (ref)	107 (14.5)	644 (85.7)	1	1
≥25 kg/m^2^	129 (31.2)	284 (68.8)	**2.19 (1.74–2.74)**	**1.93 (1.53**–2.44)^***^
Drinking alcohol habit				
No/occupational (ref)	3 (15.2)	518 (84.8)	1	1
Frequently	143 (25.9)	410 (25.9)	**1.69 (1.34–2.14)**	**1.34 (1.11**–1.76)^**^

*P < 0.05 in bold; CPR, crude prevalence ratio; CI, confidence Interval; ref, 1 reference category; APR, adjusted prevalence ratio; kg/m^2^, kilogram per meter square, h/day, hour per day*.

*The cut-point for high sedentary time ≥ 8 h per day*.

*Statistically significant:

* *P-value < 0.05*,

** *P-value < 0.01*,

*** *P-value < 0.001*.

## Discussion

The study found that about one-fifth of the working adults reported high sedentary behavior. The overall reported mean sedentary behavior time was 5.9 h/day. A large portion of the high sedentary behavior was due to leisure activities. High sedentary behavior was higher among persons in the older age group, in those involved in non-manual work, among frequent khat users, and regular alcohol drinkers, and in those with high body mass index.

The prevalence of high sedentary behavior observed in this study was within the range reported from low-and middle-income countries (25, 35). Our study showed that the mean (±SD) sedentary behavior was 5.9 (±2.1) hours per day, despite 51% of the study participants meeting WHO physical activity recommendations. The mean sedentary behavior is comparable with previous reports among university employees in low-and middle-income countries (14, 15, 36), and urban civil-servants (37). Our findings show that high sedentary behavior is prevalent in working adults in low-income countries. This might be due to labor-saving technology which is increasing the amount of high sedentary behavior. It might also be speculated that being employed increases levels of social connectedness, which may lead to high sedentary behavior and less opportunities for leisure time physical activity. Another consideration is that typical jobs in lower and middle income countries often involves non-manual labor, thus resulting in less physical activity and high sedentary behavior.

The fact that sedentary behavior occurred more often in leisure time than at work, which is consistent with previous studies (12, 14). This might be due to the increased TV viewing, computer use, video games, and social media engagements which has become more common these days (38). In addition, working and educated adults are more privileged to have access to technological advances (39). Our data were in agreement with research conducted in Western countries (35), demonstrating that working adults were more likely to be highly sedentary in China and Ghana. In urban centers of low-and-middle income countries, a more Western lifestyle may be evident, such as the use of more motorized transport, less labor-demanding jobs, and physically undemanding, mostly screen-based leisure, which may account for the higher sedentary levels in working adults in these settings.

Sedentary behavior was higher among the older age in line with the previous literature in low-and-middle income countries (25, 27). Moreover, evidence also showed that as age progresses physical activity and mobility decrease due to either loss of energy or disorders/illness that compromise physical fitness such as arthritis and musculoskeletal pain (40, 41).

Our finding related to higher sedentary behavior among those engaged in non-manual work is related to the nature of their work that requires prolonged sitting (42). Non-manual works these days involve using a computer that potentially requires minimal or no physical activity (43). In such occupations, sedentary behavior may not affect productivity (44, 45).

Our study shows that sedentary behavior was associated with frequent consumption of alcohol and khat. Similar findings were reported previously (46, 47). The use of alcohol and especially Khat involves prolonged sitting. Khat chewing/consumption in eastern Ethiopia is ceremonial and lasts up to 6 h at a time. Frequent attendance to such Khat chewing ceremony increases the time spent in sedentary status (48).

High body mass index was significantly associated with a high prevalence of sedentary behavior in line with previous studies in sub-Saharan Africa (49). The consistency with past studies confirms that the hypothesis of individuals with high BMI may go into a vicious cycle whereby a decrease in mobility reduces body energy expenditure leading to weight gain and then weight gain reduces mobility and leads to the adoption of sedentarism (50).

Although dichotomous analyses of sedentary behavior are regarded as more informative (51), there is no consensus on a single cut-off for sedentary behavior; thus we used a cut-off point from recent publications (6, 26, 52). Thus comparing our findings must be done cautiously as researchers use different cut-off points and diverse sedentary behavior assessment tools (53). The use of different tools can significantly influence the results; a self-reported domain-specific sedentary behavior questionnaires give substantially higher sedentary time estimates than using single-item sedentary time questions.

### Limitation and Strengths

The main limitation of this study includes reliance on self-reporting, which is less accurate than sedentary behavior assessed using devices available for this purpose (54). Self-report is likely to be affected by recall bias and social desirability bias that potentially underestimates sedentary behavior. Furthermore, the study did not consider ecological variables such as psychosocial factors, organizational/community, environmental, and policy factors related to sedentary behavior due to resource constraints. Lastly, this study may not be generalizable to all working adults in eastern Ethiopia as the study population was drawn only from one institution. However, the strengths of this study include a large sample size and the use of standardized data collection tools. Moreover, this study was the first of its kind among university employees in Ethiopia and can be fairly generalized for this category of workers in areas where there are contextual working adults.

## Conclusions

About one-fifth of Ethiopia university workers reported high sedentary behavior, and leisure sedentary time was the predominant domain. Factors associated with high sedentary behavior among Ethiopia university workers were older age, being a non-manual worker, regular alcohol drinker, frequent khat-chewer, and overweight/obesity. Further study is needed by using device measurement and a representative population to check the reputability of self-report sedentary behavior and to reduce the source of errors and bias.

## Data Availability Statement

The raw data supporting the conclusions of this article will be made available by the authors, without undue reservation.

## Ethics Statement

The studies involving human participants were reviewed and approved by Haramaya University, College of Health and Medical Sciences Institutional Health Research Ethics Review Committee (IHRERC). The patients/participants provided their written informed consent to participate in this study.

## Author Contributions

All authors made substantial contributions to conception and design, acquisition of data, or analysis and interpretation of data, took part in drafting the article or revising it critically for important intellectual content, gave final approval of the version to be published, agreed to be accountable for all aspects of the work, read, and approved the final manuscript.

## Funding

This study was supported by Haramaya University (Grant # HURG-2017-02-02-05) only for data collection purpose. The university has no role on deciding the study design, data collection, analysis and interpretation, and manuscript writing and publication. Addis Continental Institute of Public Health provided technical assistance.

## Conflict of Interest

The authors declare that the research was conducted in the absence of any commercial or financial relationships that could be construed as a potential conflict of interest.

## Publisher's Note

All claims expressed in this article are solely those of the authors and do not necessarily represent those of their affiliated organizations, or those of the publisher, the editors and the reviewers. Any product that may be evaluated in this article, or claim that may be made by its manufacturer, is not guaranteed or endorsed by the publisher.

## Abbreviations

BMI: body Mass Index
MET: metabolic equivalent of task
SB: sedentary behavior
SBQ: sedentary behavior questioner
SD: standard deviation
WHO: World Health Organization
NCDs: non-communicable diseases.
